# Supplementary figures and images for: SMC5/6-mediated plasmid silencing is directed by SIMC1–SLF2 and antagonized by the SV40 large T antigen
Source: eLife. 2025 Nov 26;14:RP106815. doi: 10.7554/eLife.106815 (PMC12656486; doi:10.7554/eLife.106815)

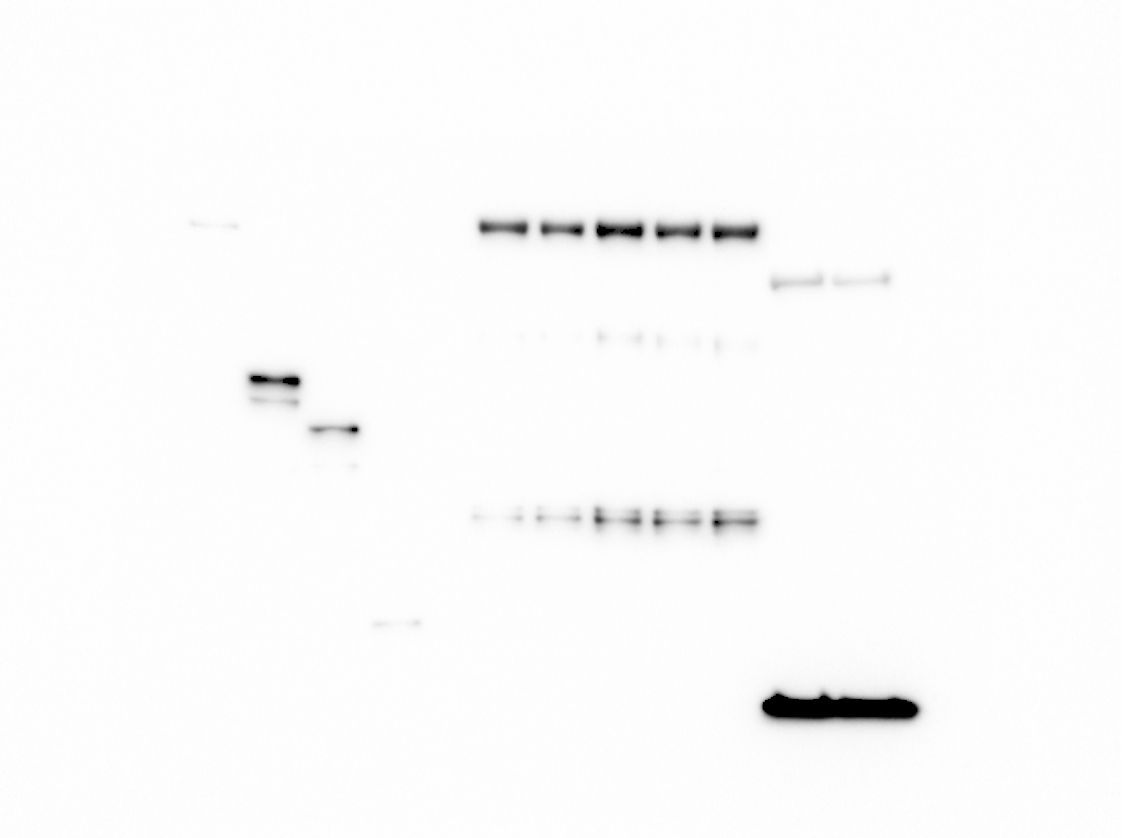

Supplement: Figure 1—source data 1. [file elife-106815-fig1-data1.zip › raw/Figure 1C_GFP IP.jpg]

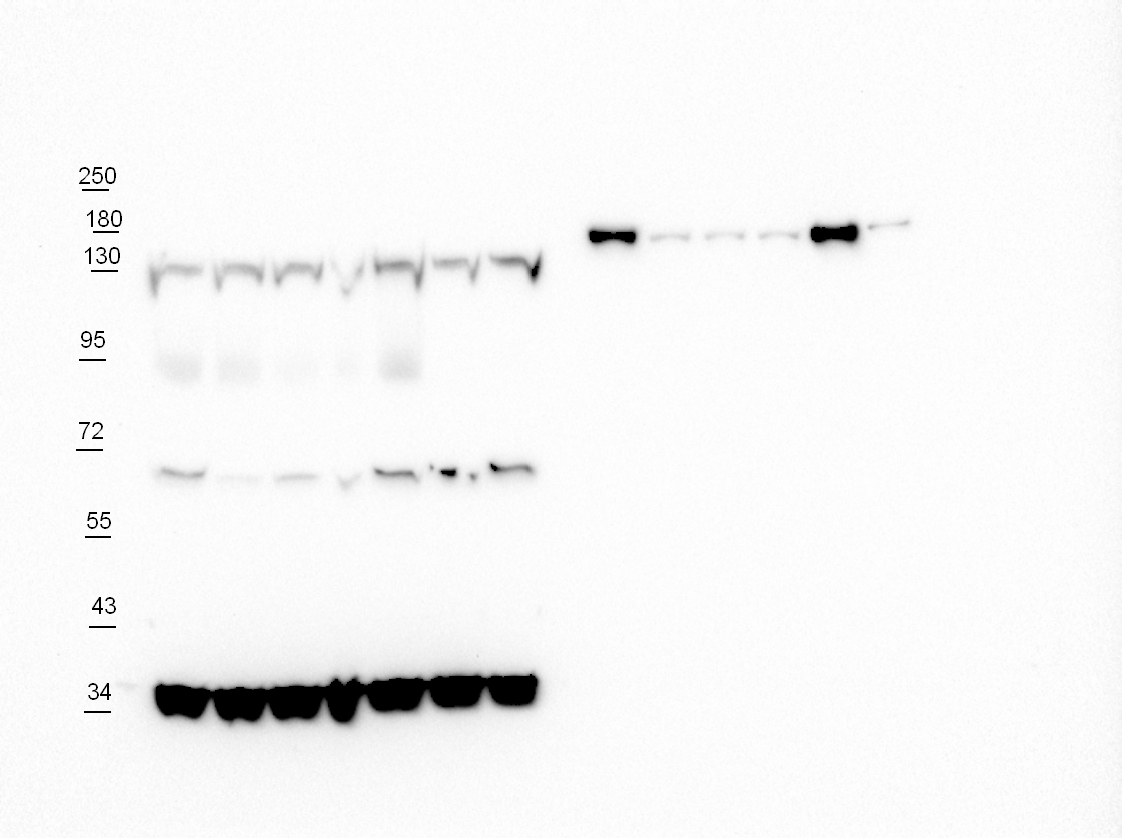

Supplement: Figure 1—source data 1. [file elife-106815-fig1-data1.zip › raw/Figure 1C_SMC6.jpg]

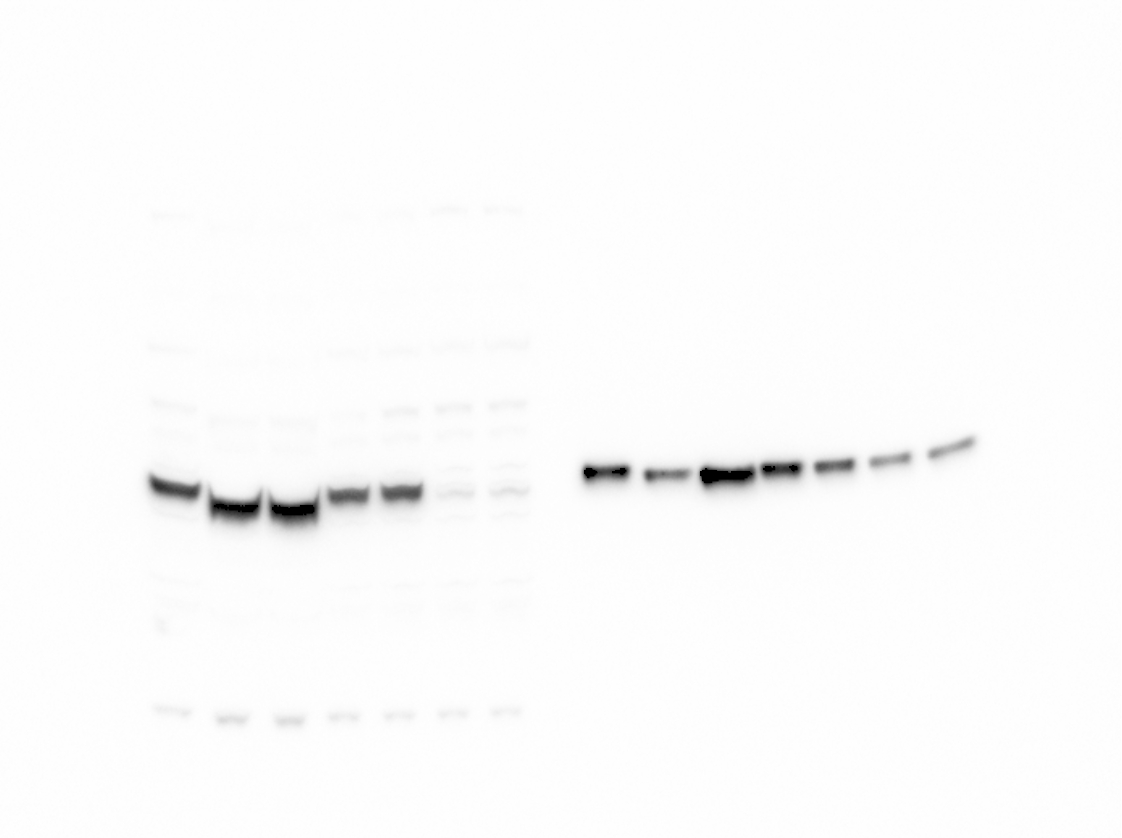

Supplement: Figure 1—source data 1. [file elife-106815-fig1-data1.zip › raw/Figure 1C_FLAG.jpg]

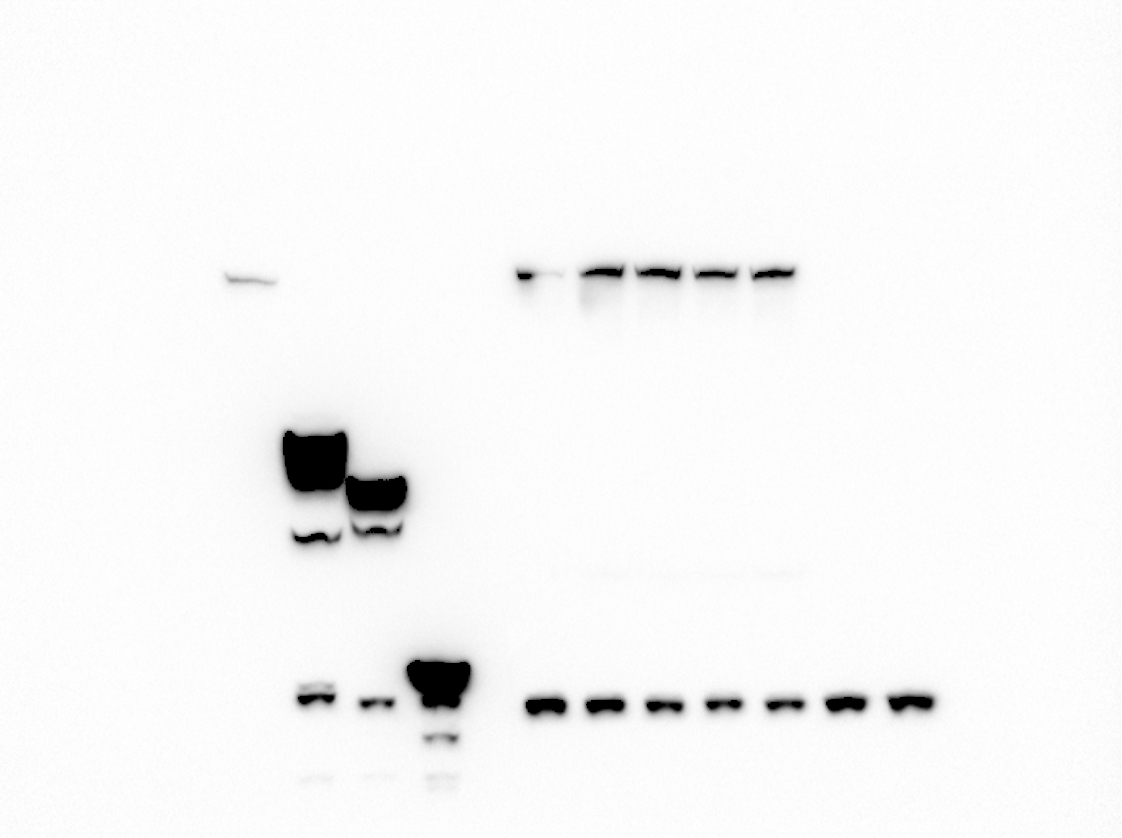

Supplement: Figure 1—source data 1. [file elife-106815-fig1-data1.zip › raw/Figure 1C_GFP input.jpg]

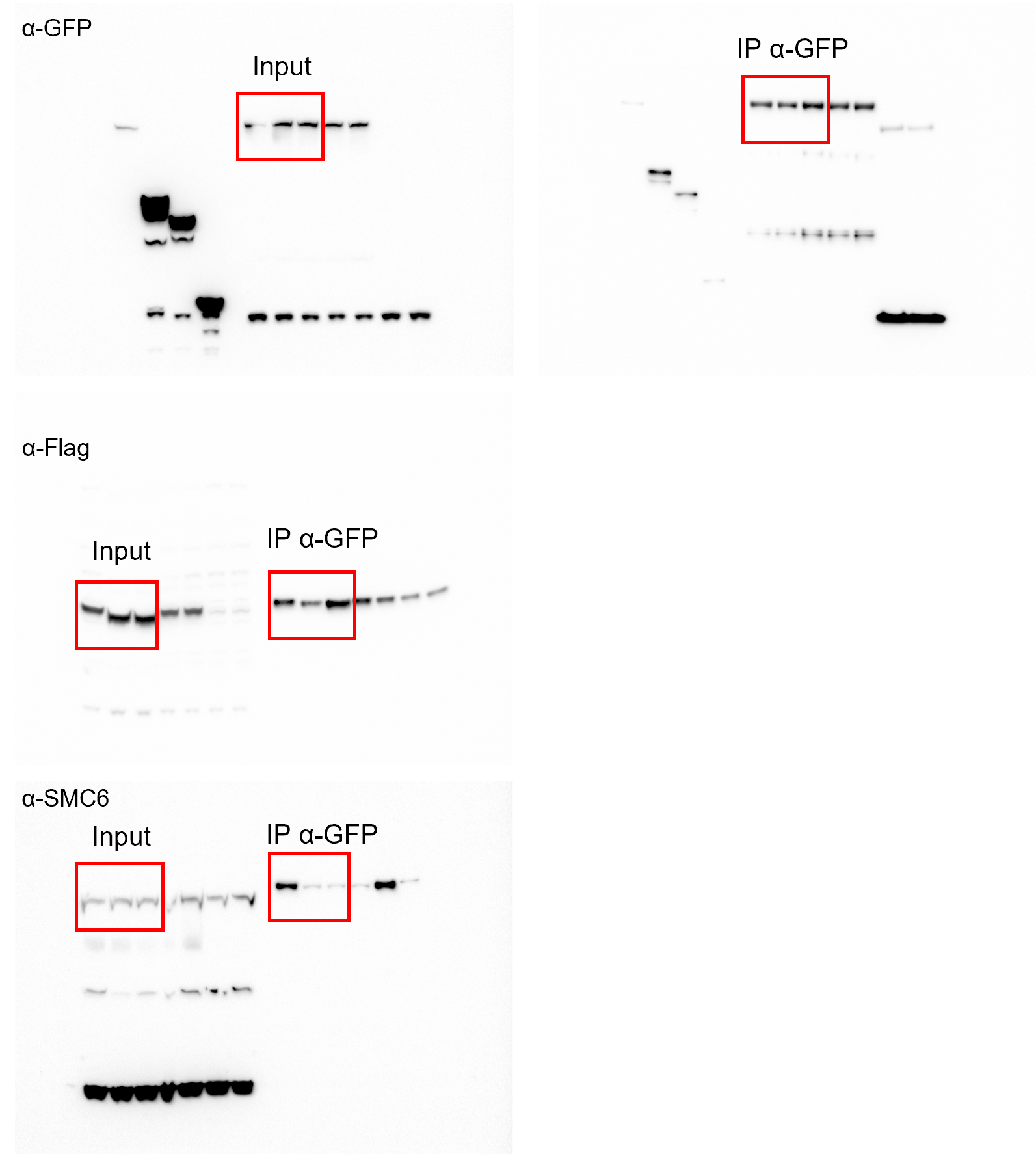

Supplement: Figure 1—source data 1. [file elife-106815-fig1-data1.zip › labelled/Figure 1C_labeled.tif]

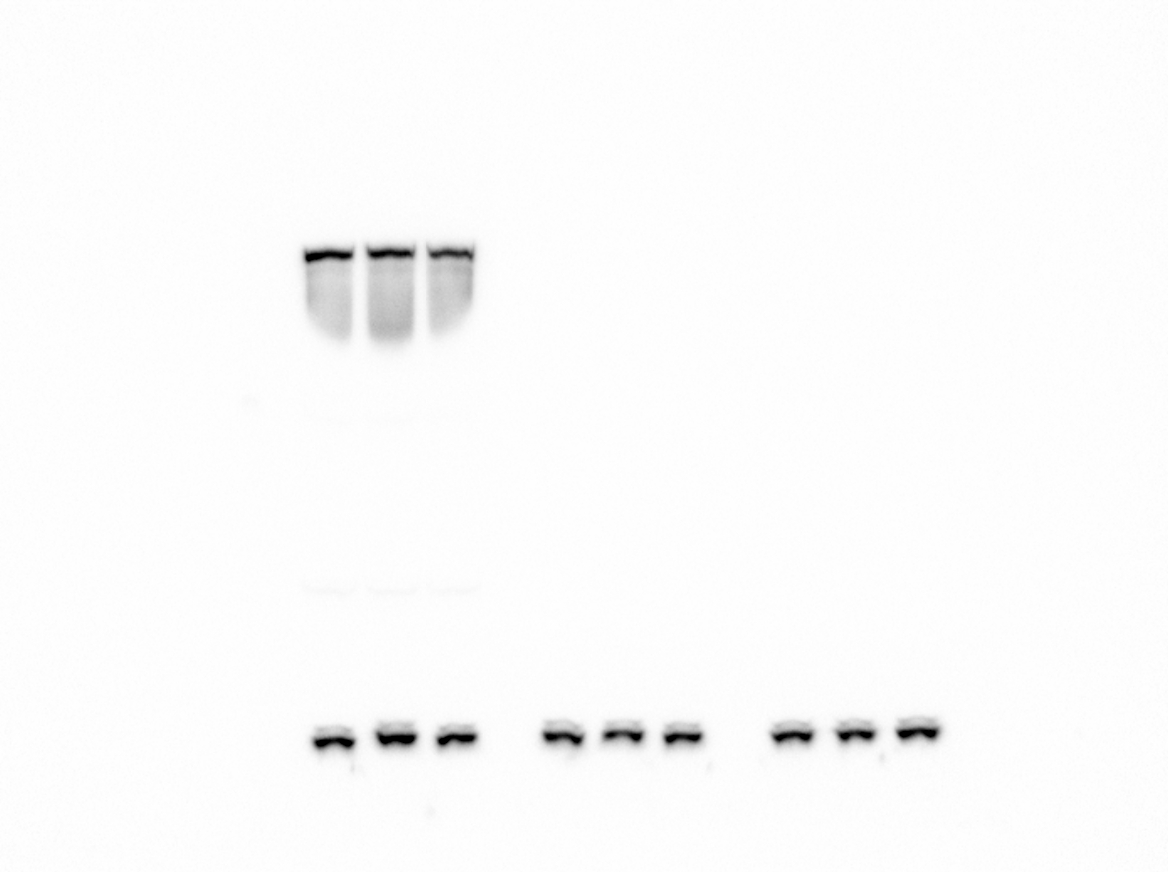

Supplement: Figure 1—figure supplement 1—source data 1. [file elife-106815-fig1-figsupp1-data1.zip › raw/Figure 1-figure supplement 1A_GFP input.jpg]

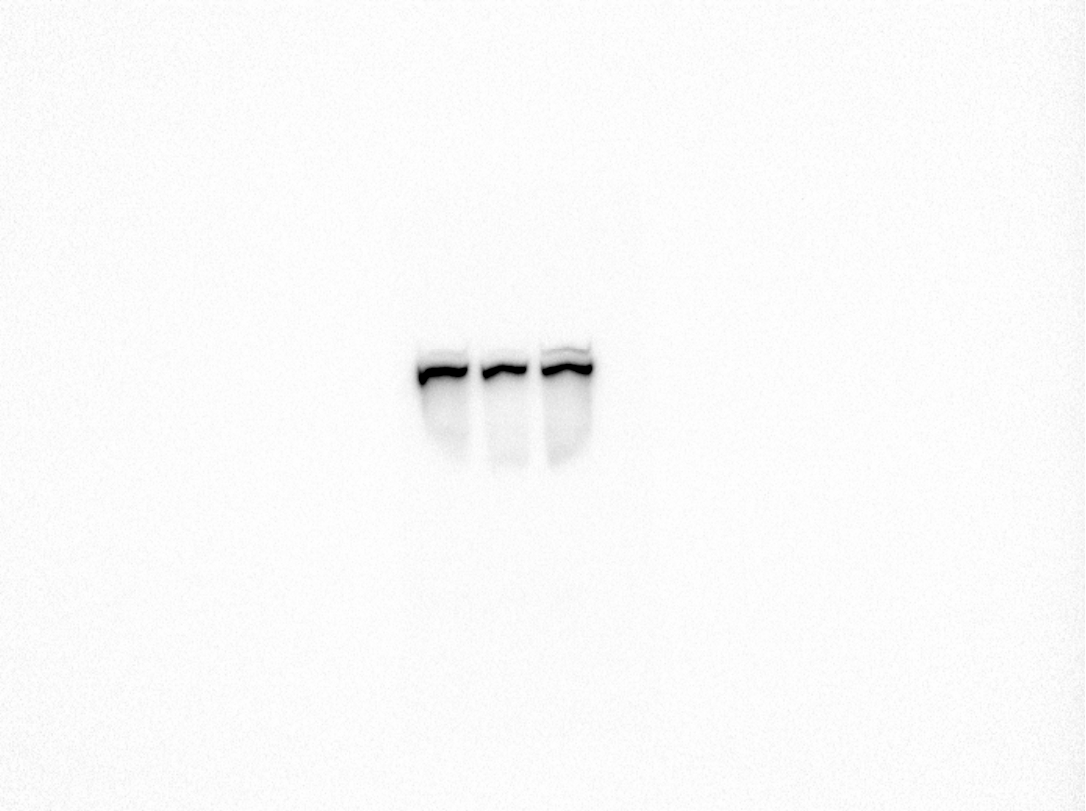

Supplement: Figure 1—figure supplement 1—source data 1. [file elife-106815-fig1-figsupp1-data1.zip › raw/Figure 1-figure supplement 1A_SMC6 input.jpg]

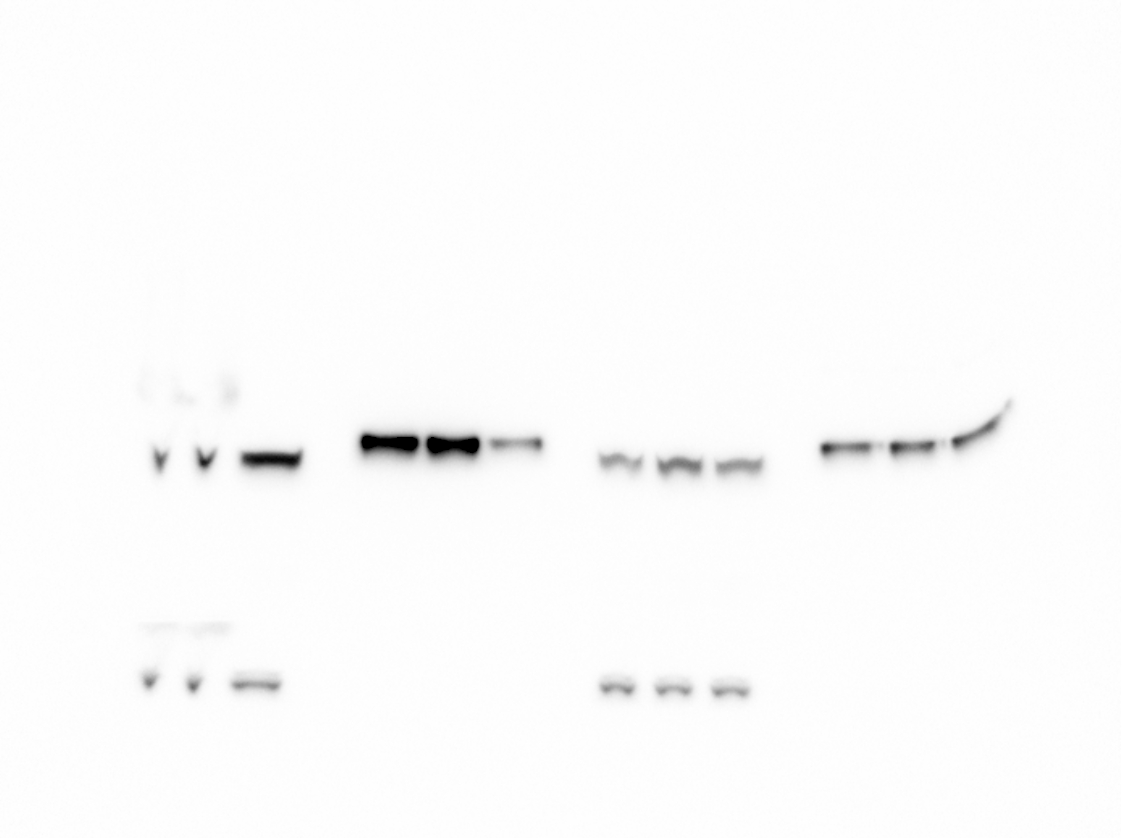

Supplement: Figure 1—figure supplement 1—source data 1. [file elife-106815-fig1-figsupp1-data1.zip › raw/Figure 1-figure supplement 1A_FLAG IP.jpg]

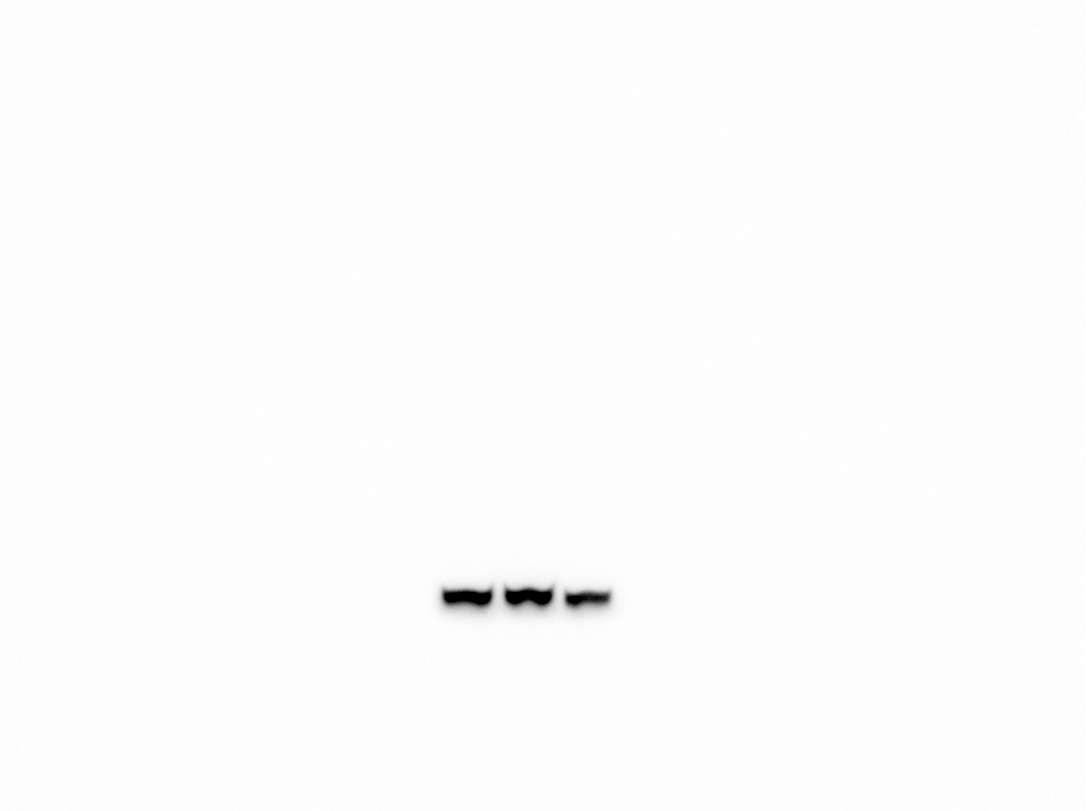

Supplement: Figure 1—figure supplement 1—source data 1. [file elife-106815-fig1-figsupp1-data1.zip › raw/Figure 1-figure supplement 1A_FLAG input.jpg]

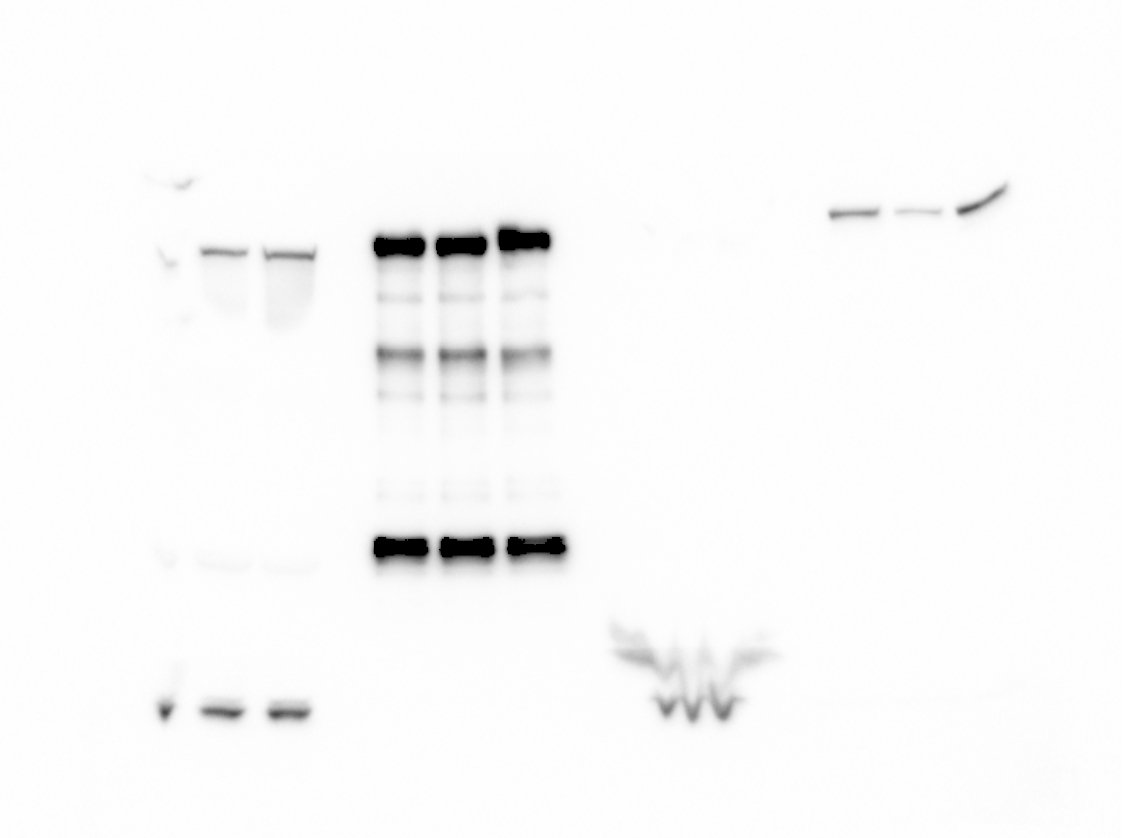

Supplement: Figure 1—figure supplement 1—source data 1. [file elife-106815-fig1-figsupp1-data1.zip › raw/Figure 1-figure supplement 1A_GFP IP.jpg]

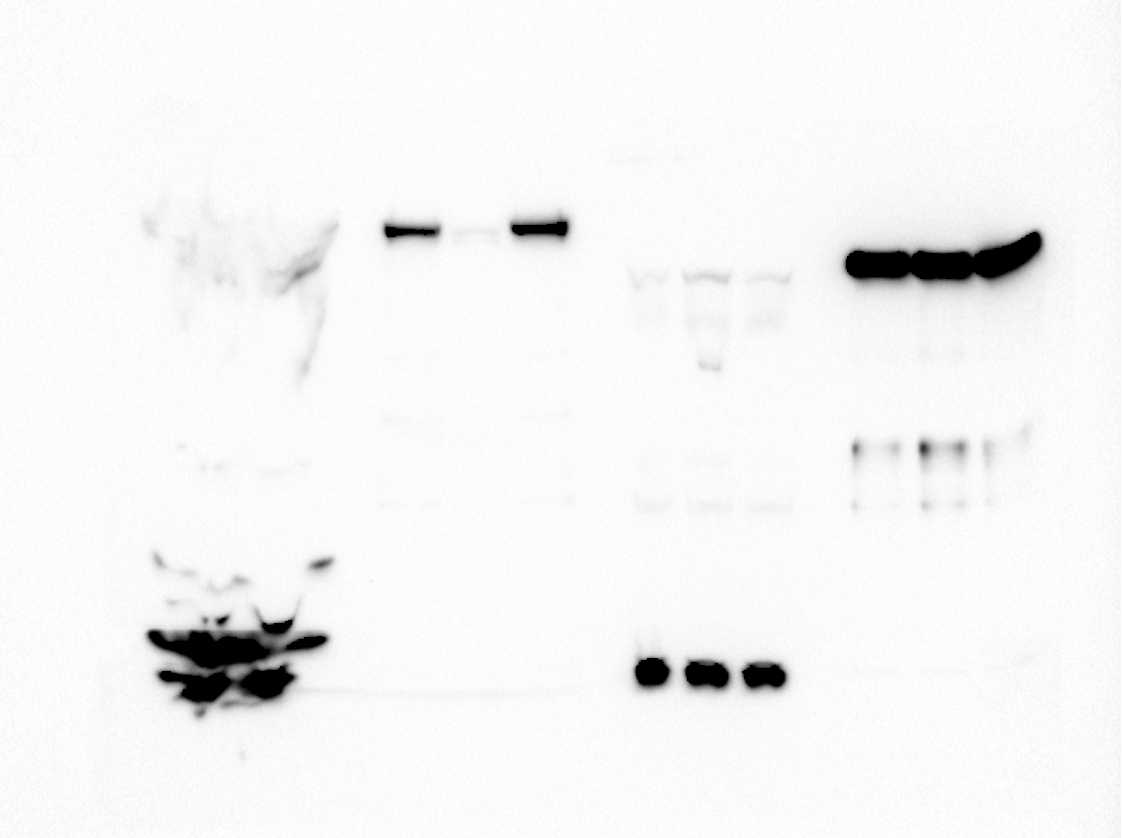

Supplement: Figure 1—figure supplement 1—source data 1. [file elife-106815-fig1-figsupp1-data1.zip › raw/Figure 1-figure supplement 1A_SMC6 IP.jpg]

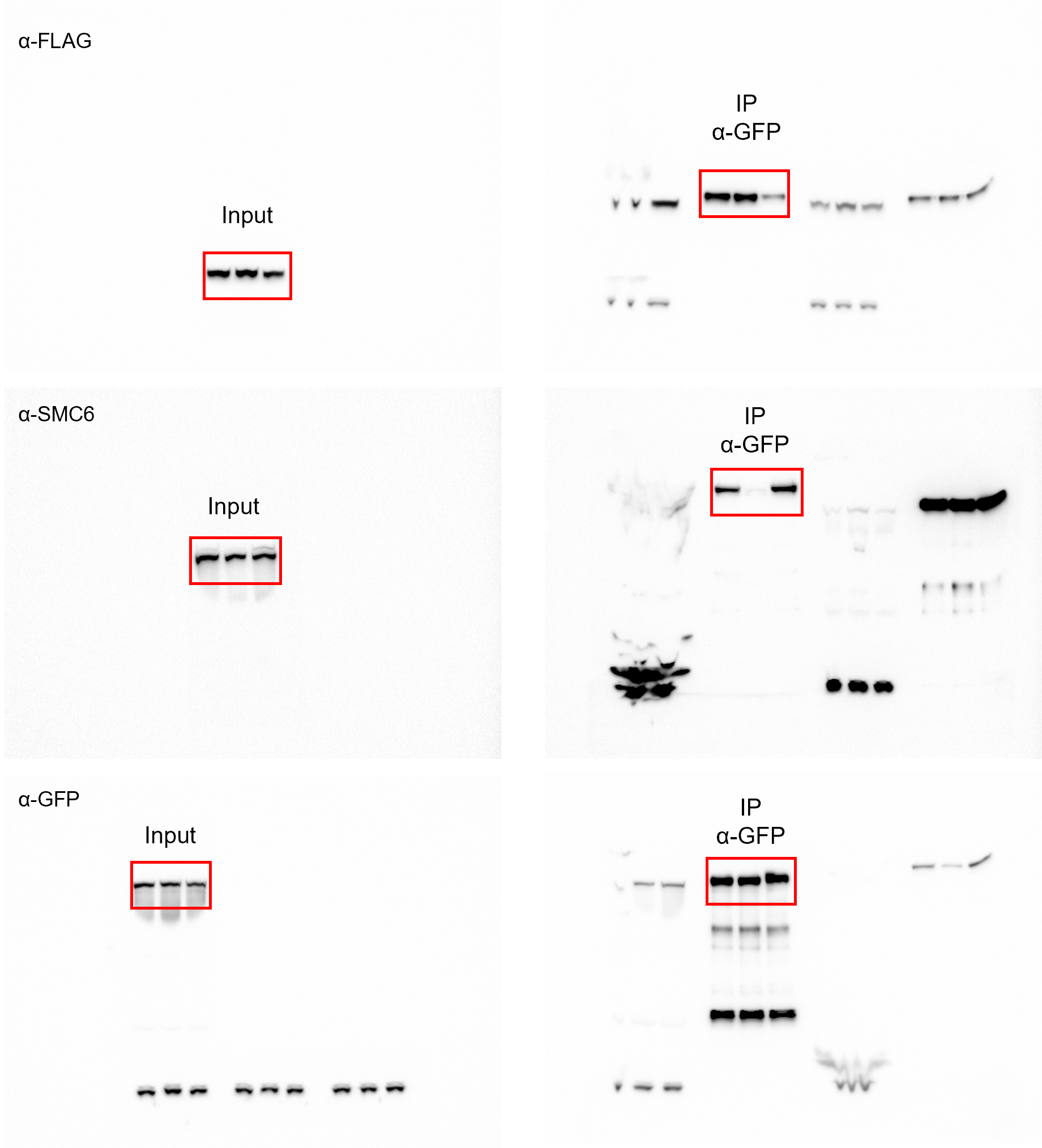

Supplement: Figure 1—figure supplement 1—source data 1. [file elife-106815-fig1-figsupp1-data1.zip › labelled/Figure 1-figure supplement 1A_labeled.tif]

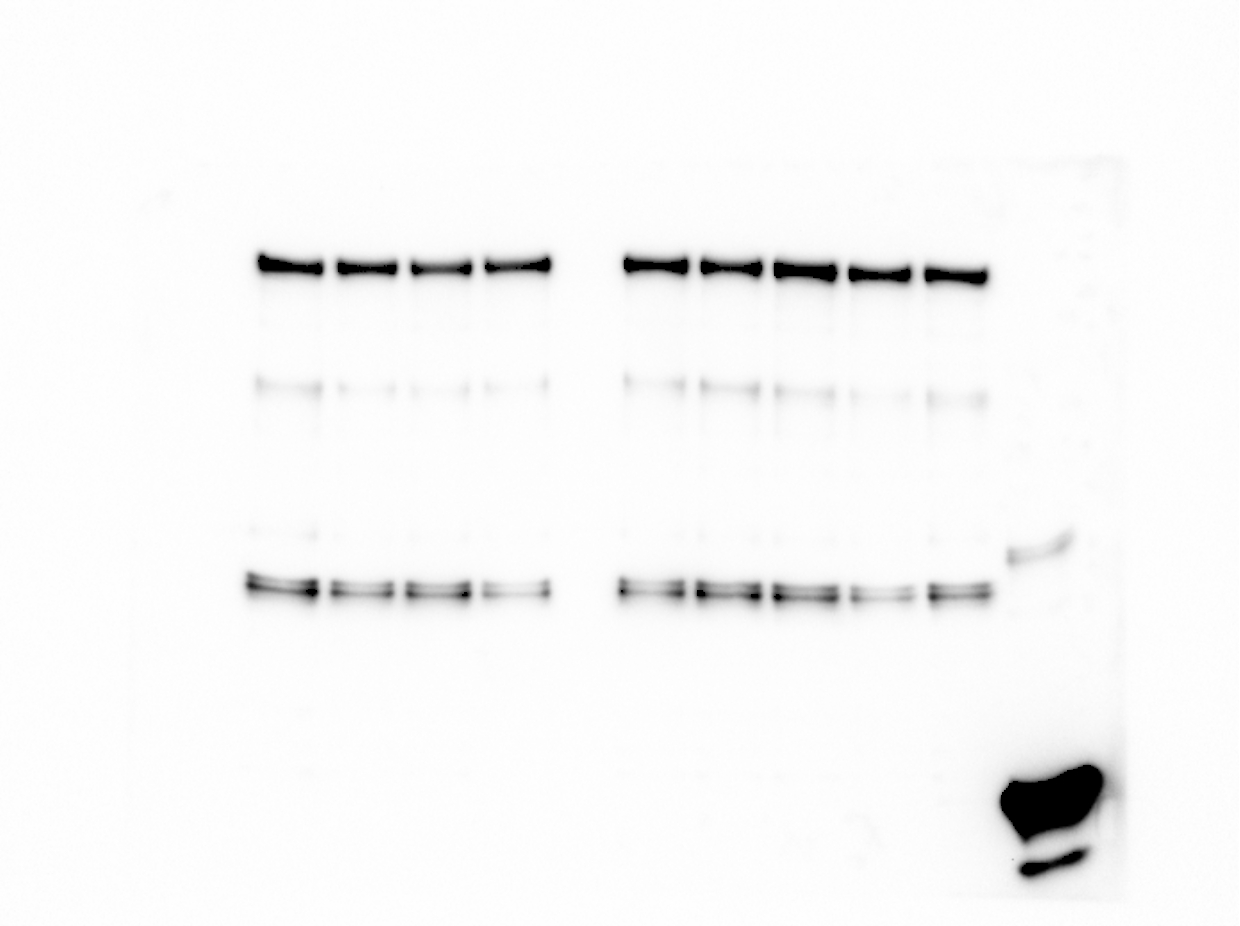

Supplement: Figure 1—figure supplement 1—source data 2. [file elife-106815-fig1-figsupp1-data2.zip › raw/Figure 1-figure supplement 1B_GFP IP.jpg]

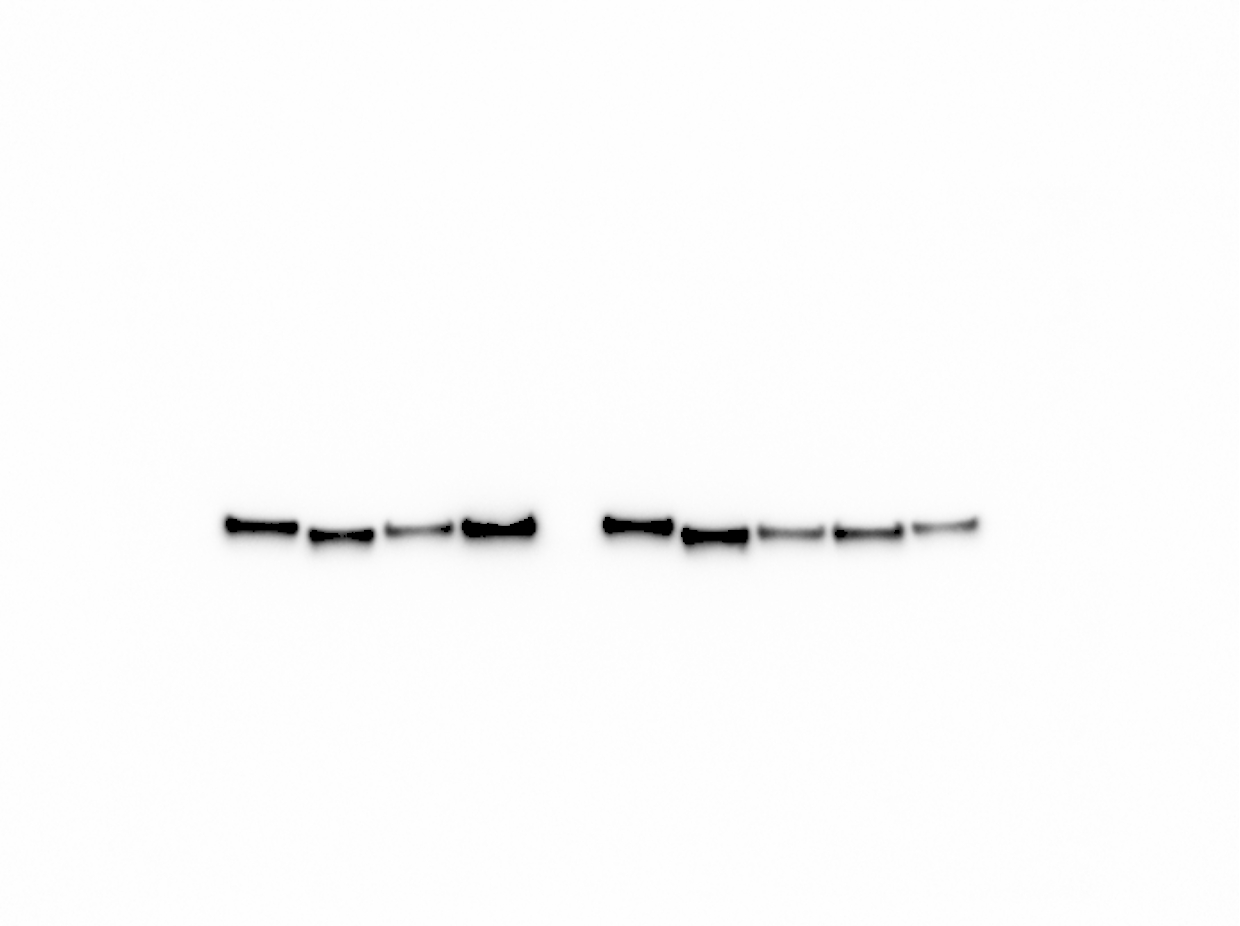

Supplement: Figure 1—figure supplement 1—source data 2. [file elife-106815-fig1-figsupp1-data2.zip › raw/Figure 1-figure supplement 1B_FLAG IP.jpg]

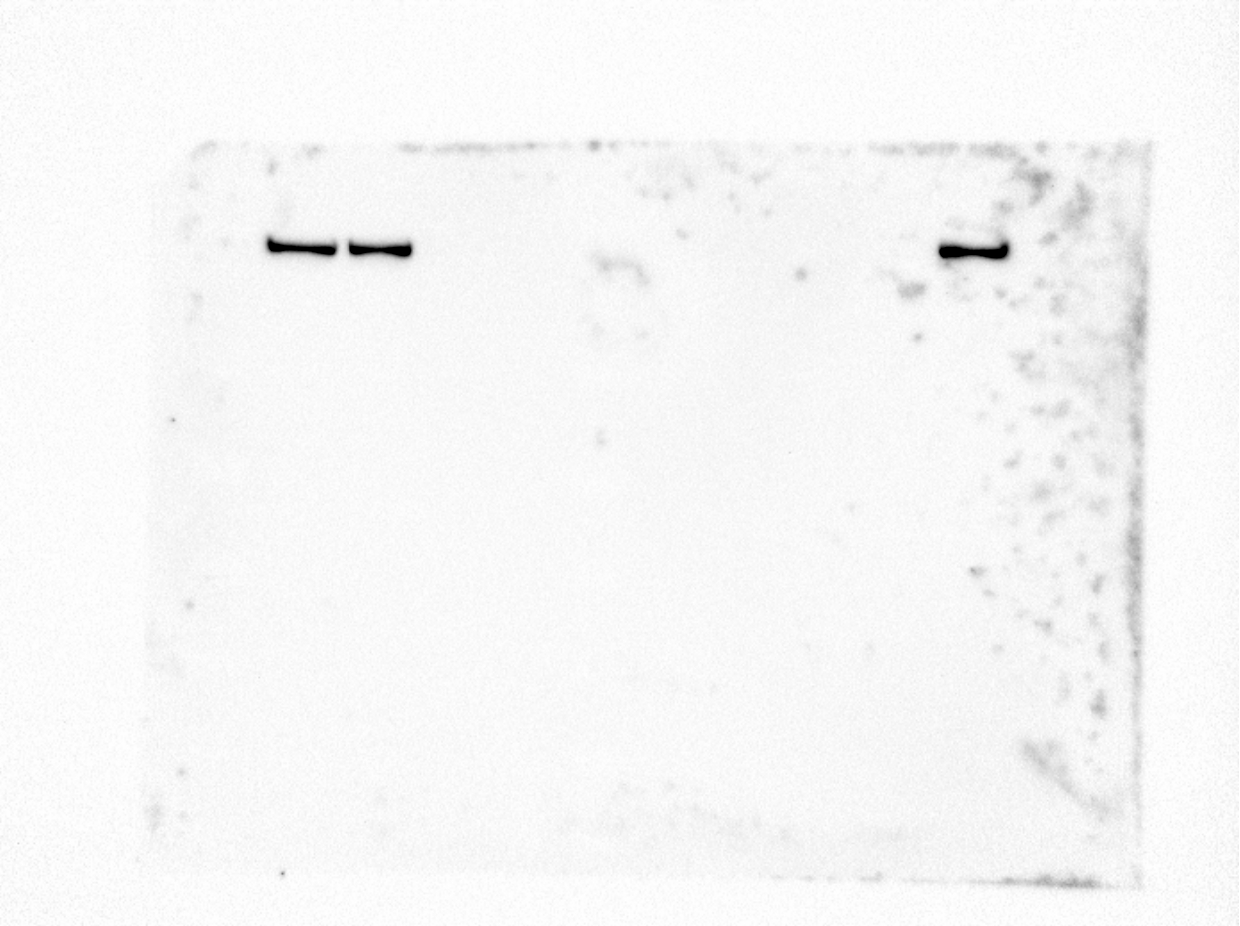

Supplement: Figure 1—figure supplement 1—source data 2. [file elife-106815-fig1-figsupp1-data2.zip › raw/Figure 1-figure supplement 1B_Myc IP.jpg]

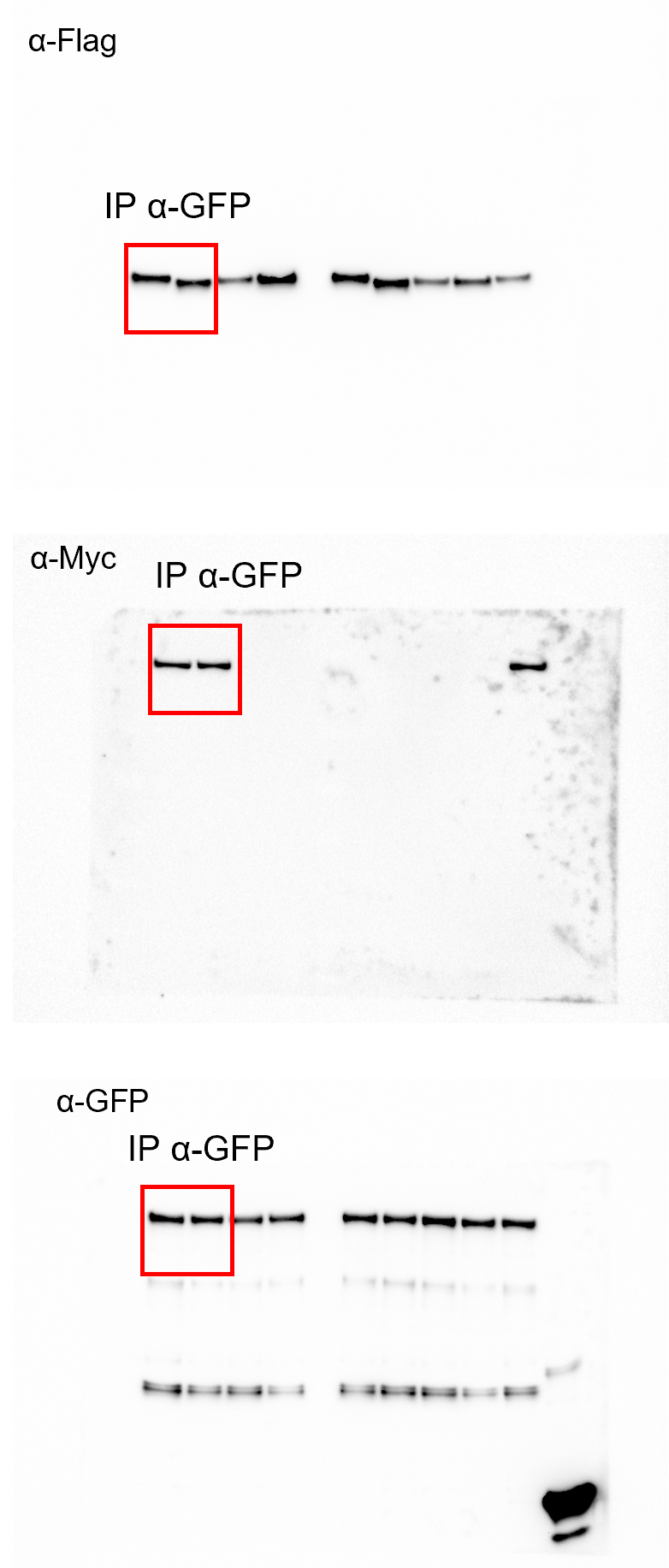

Supplement: Figure 1—figure supplement 1—source data 2. [file elife-106815-fig1-figsupp1-data2.zip › labelled/Figure 1-figure supplement 1B_labeled.tif]

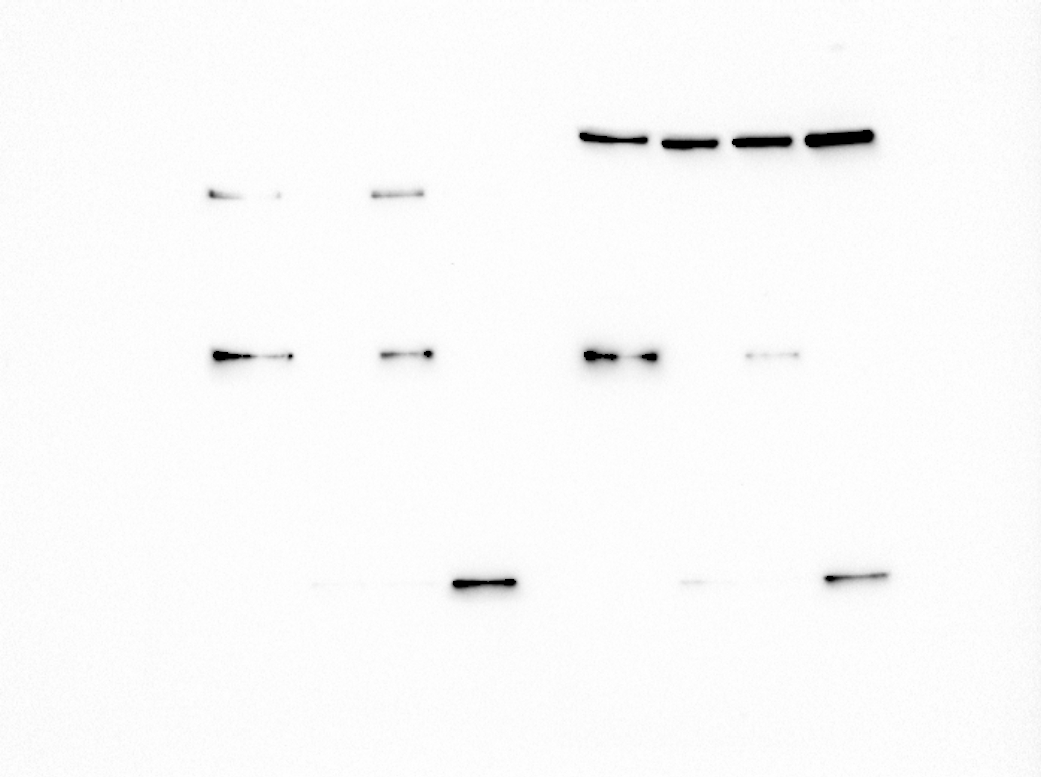

Supplement: Figure 2—source data 1. [file elife-106815-fig2-data1.zip › raw/Figure 2C_GFP IP.jpg]

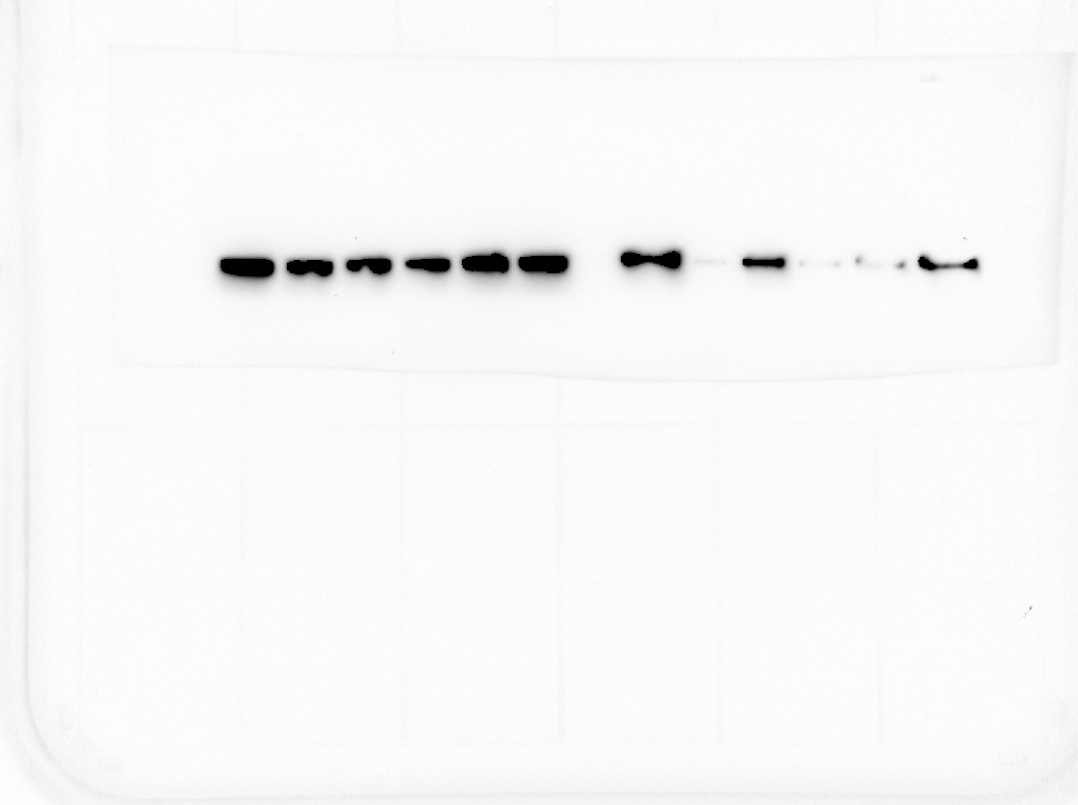

Supplement: Figure 2—source data 1. [file elife-106815-fig2-data1.zip › raw/Figure 2C_Myc input.jpg]

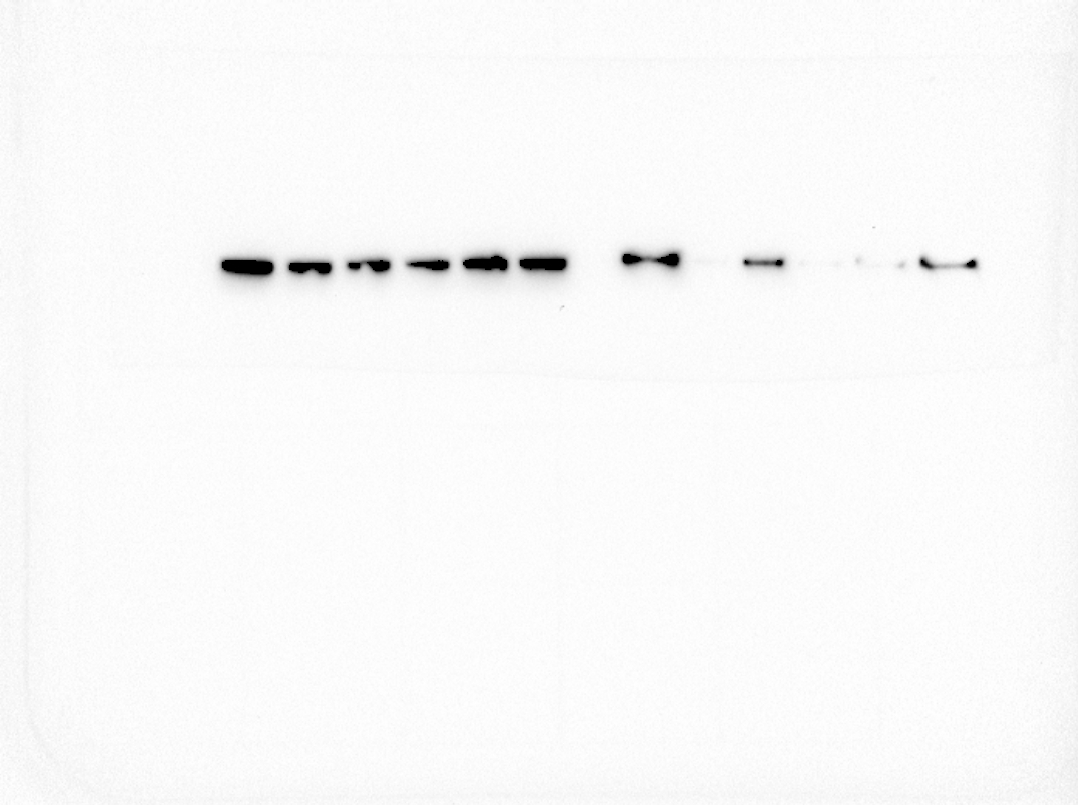

Supplement: Figure 2—source data 1. [file elife-106815-fig2-data1.zip › raw/Figure 2C_Myc IP.jpg]

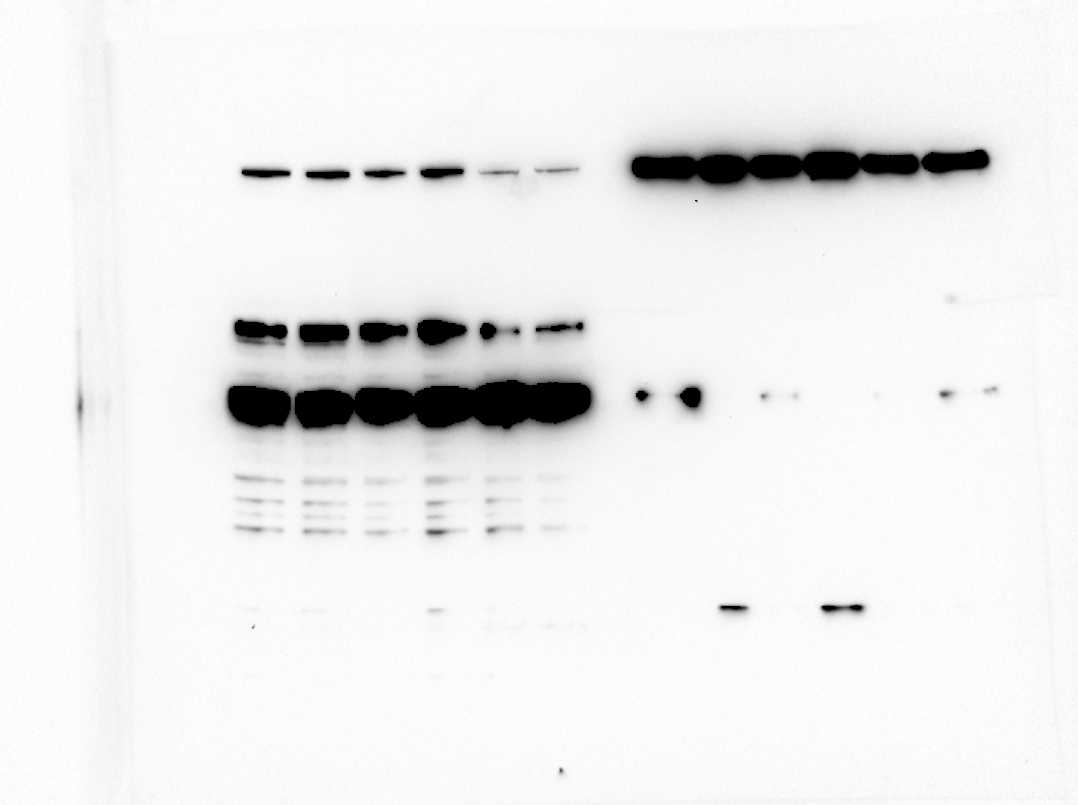

Supplement: Figure 2—source data 1. [file elife-106815-fig2-data1.zip › raw/Figure 2C_GFP input.jpg]

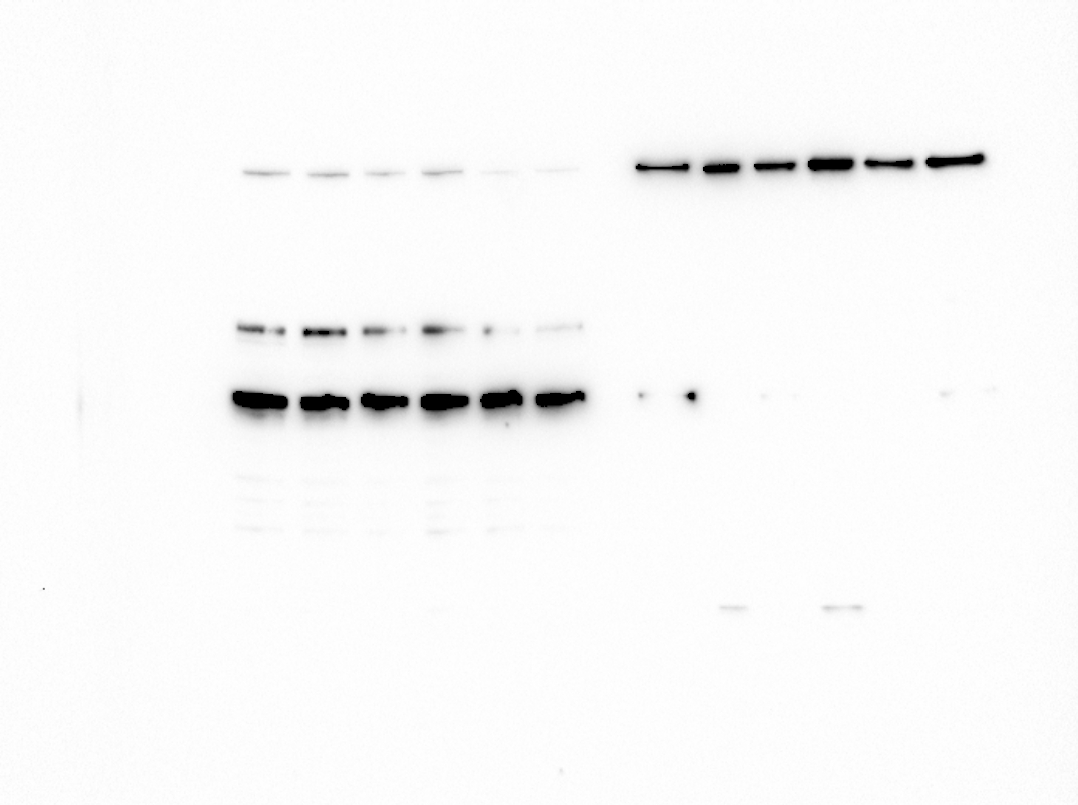

Supplement: Figure 2—source data 1. [file elife-106815-fig2-data1.zip › raw/Figure 2C_FLAG input.jpg]

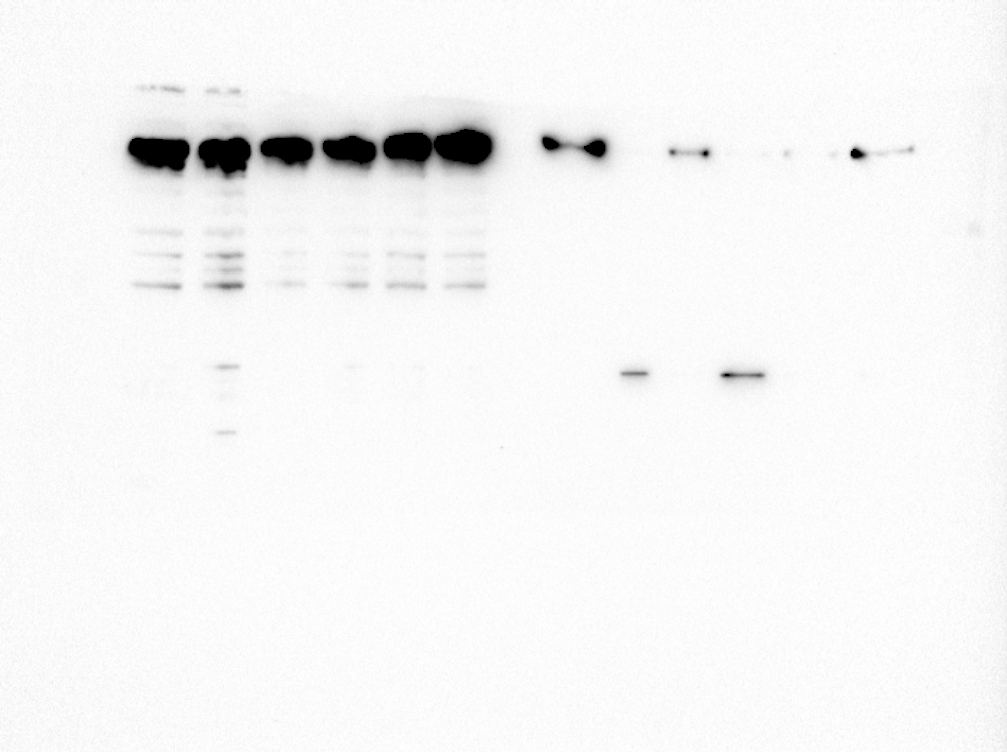

Supplement: Figure 2—source data 1. [file elife-106815-fig2-data1.zip › raw/Figure 2C_FLAG IP.jpg]

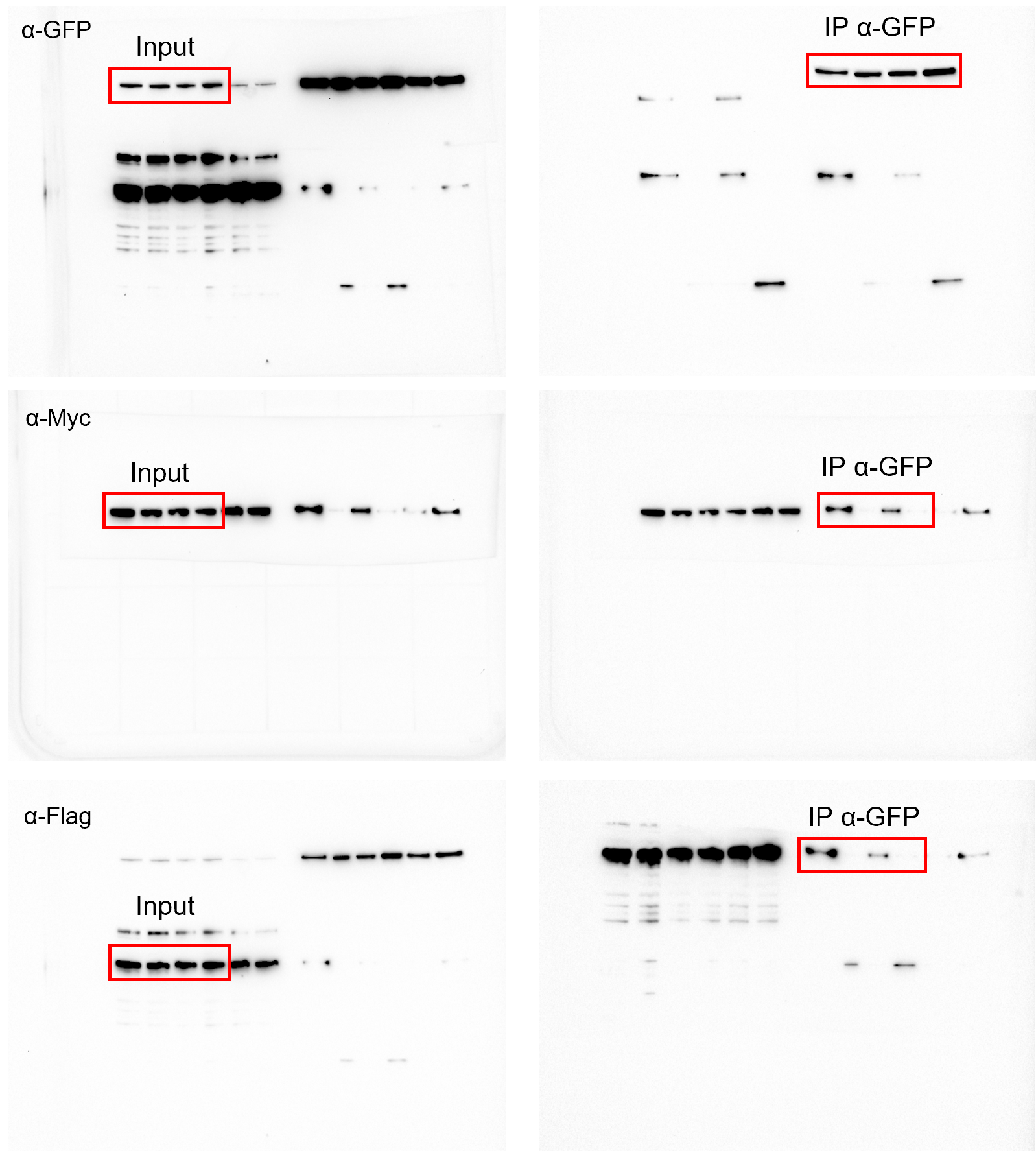

Supplement: Figure 2—source data 1. [file elife-106815-fig2-data1.zip › labelled/Figure 2C_labeled.tif]

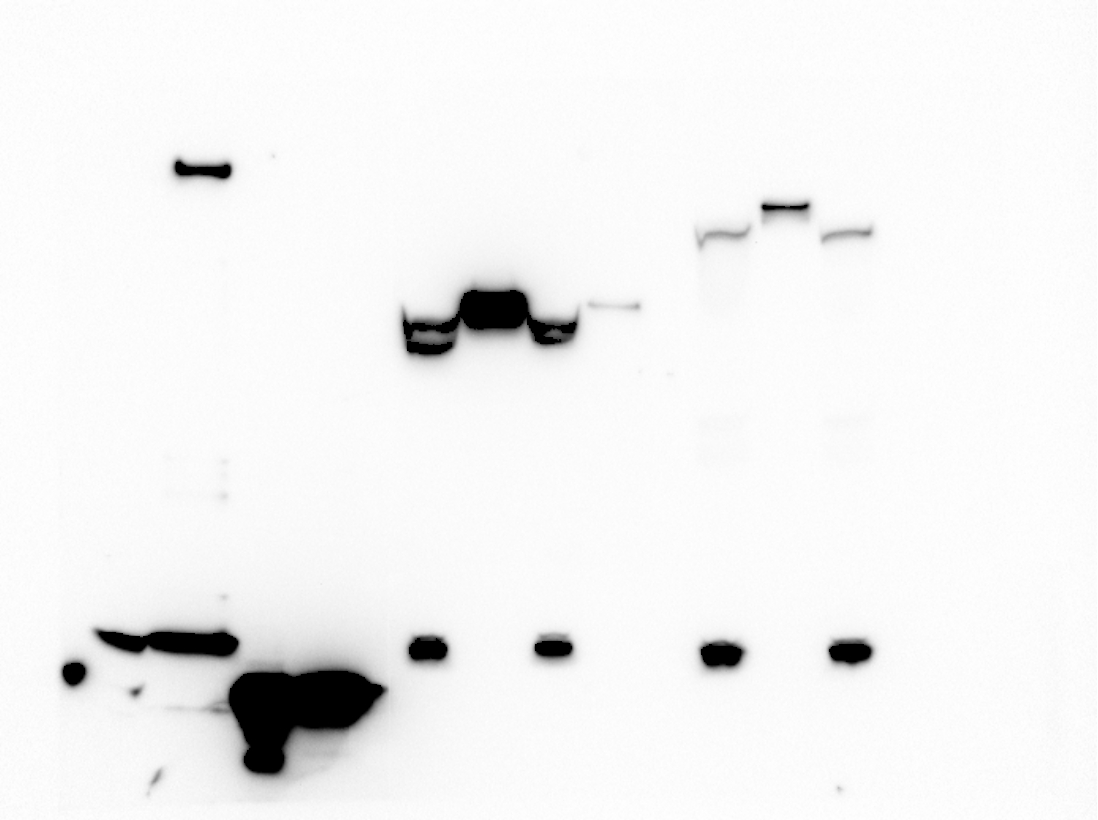

Supplement: Figure 5—source data 3. [file elife-106815-fig5-data3.zip › raw/Figure 5C_SMC6.jpg]

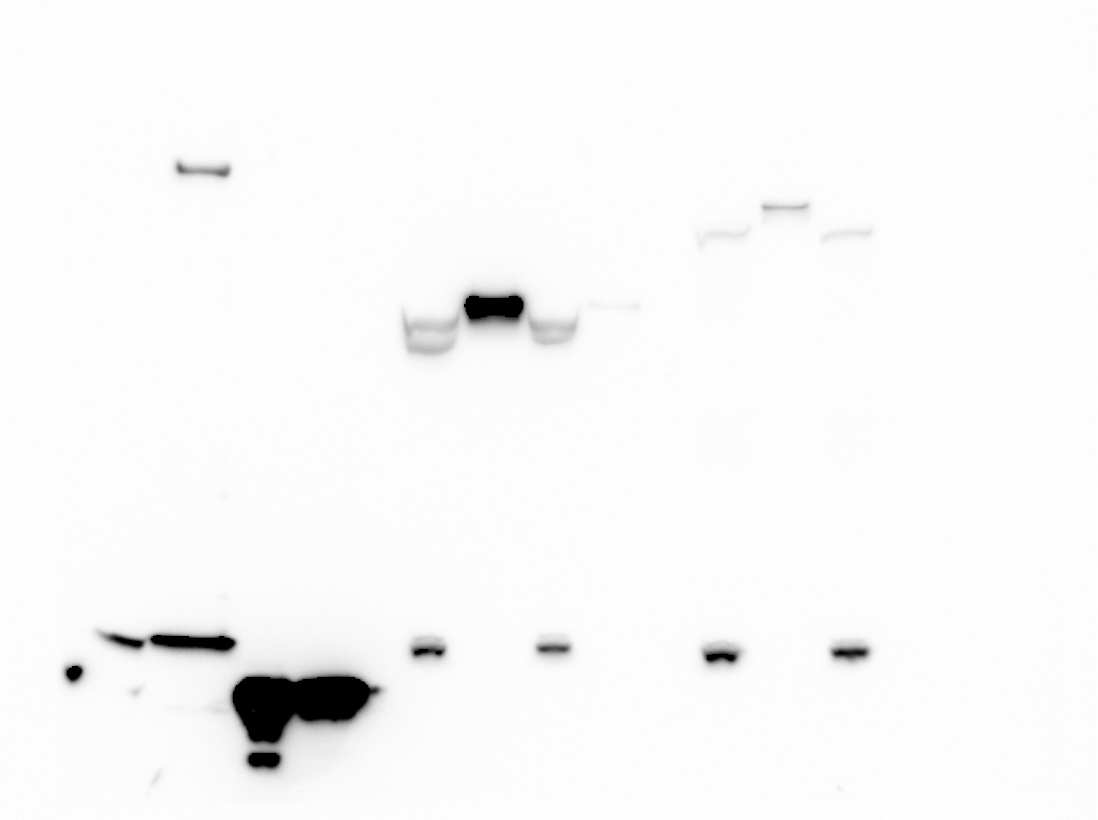

Supplement: Figure 5—source data 3. [file elife-106815-fig5-data3.zip › raw/Figure 5C_LT.jpg]

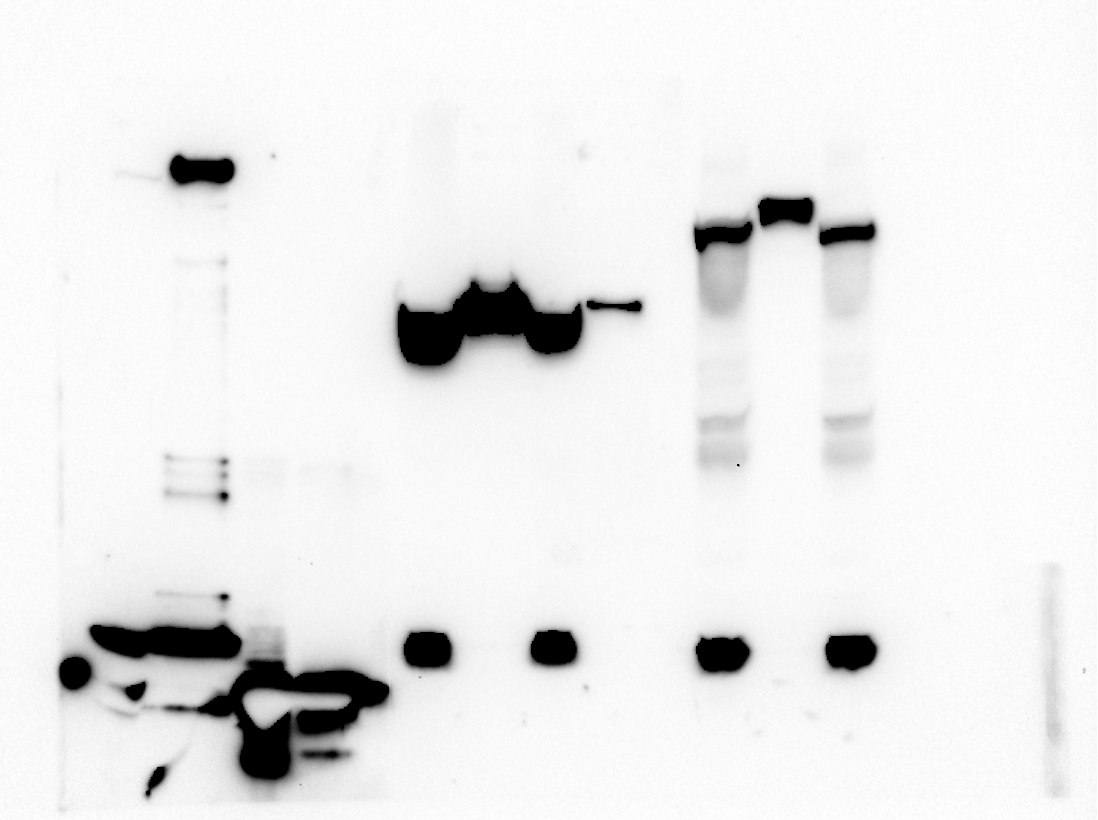

Supplement: Figure 5—source data 3. [file elife-106815-fig5-data3.zip › raw/Figure 5C_GFP1.jpg]

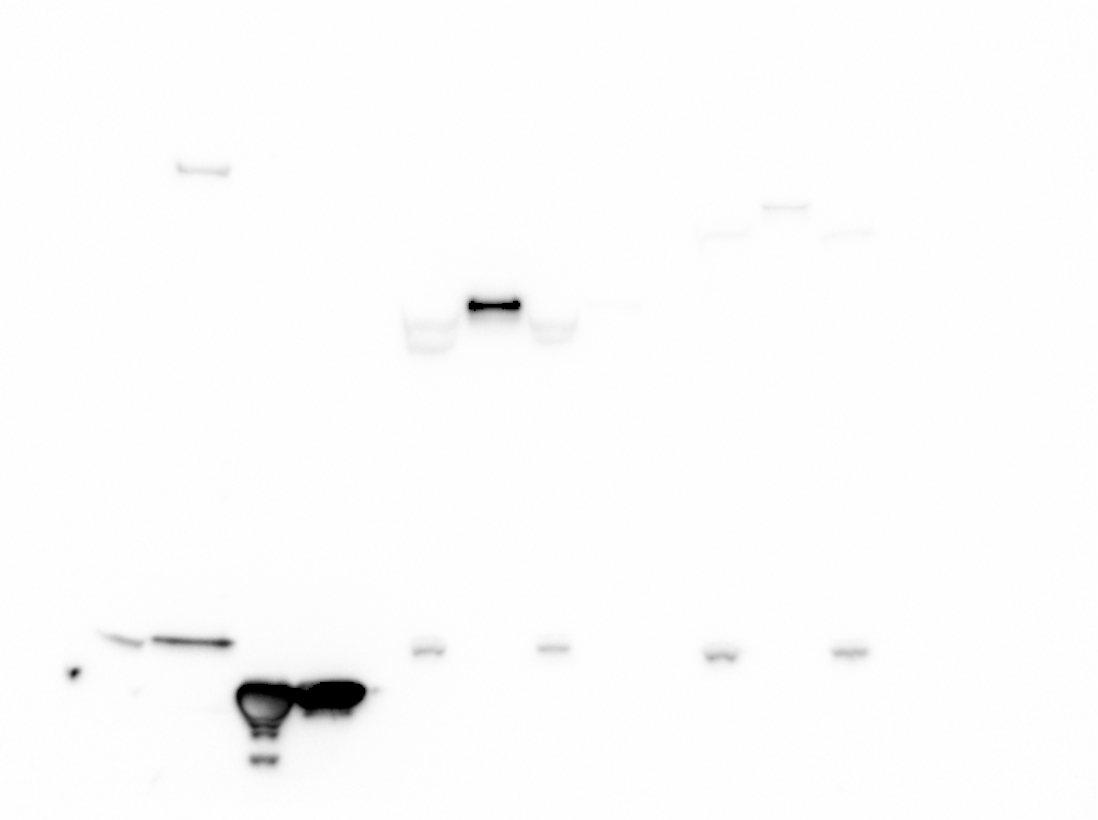

Supplement: Figure 5—source data 3. [file elife-106815-fig5-data3.zip › raw/Figure 5C_GFP 2.jpg]

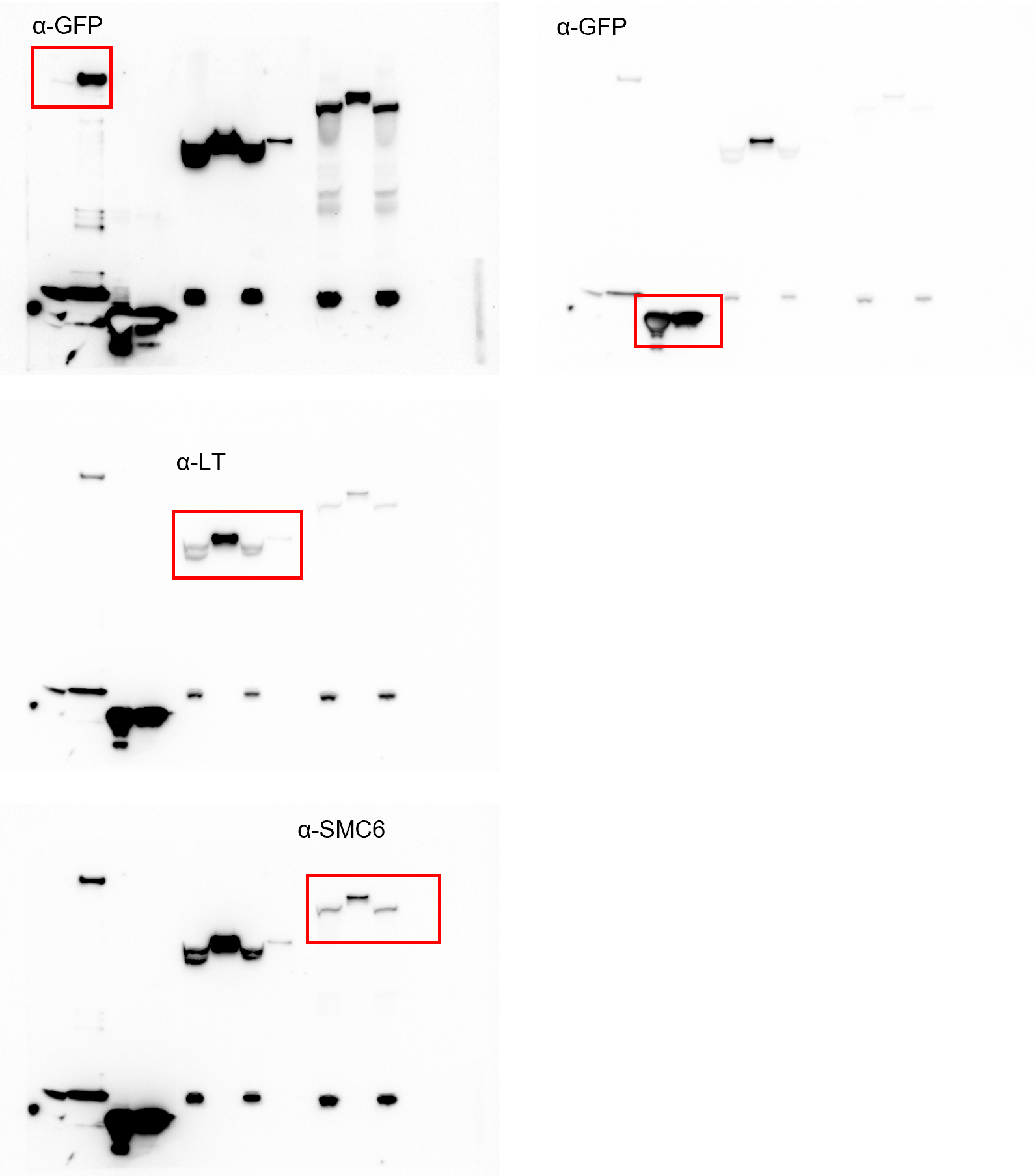

Supplement: Figure 5—source data 3. [file elife-106815-fig5-data3.zip › labelled/Figure 5C_labeled.tif]

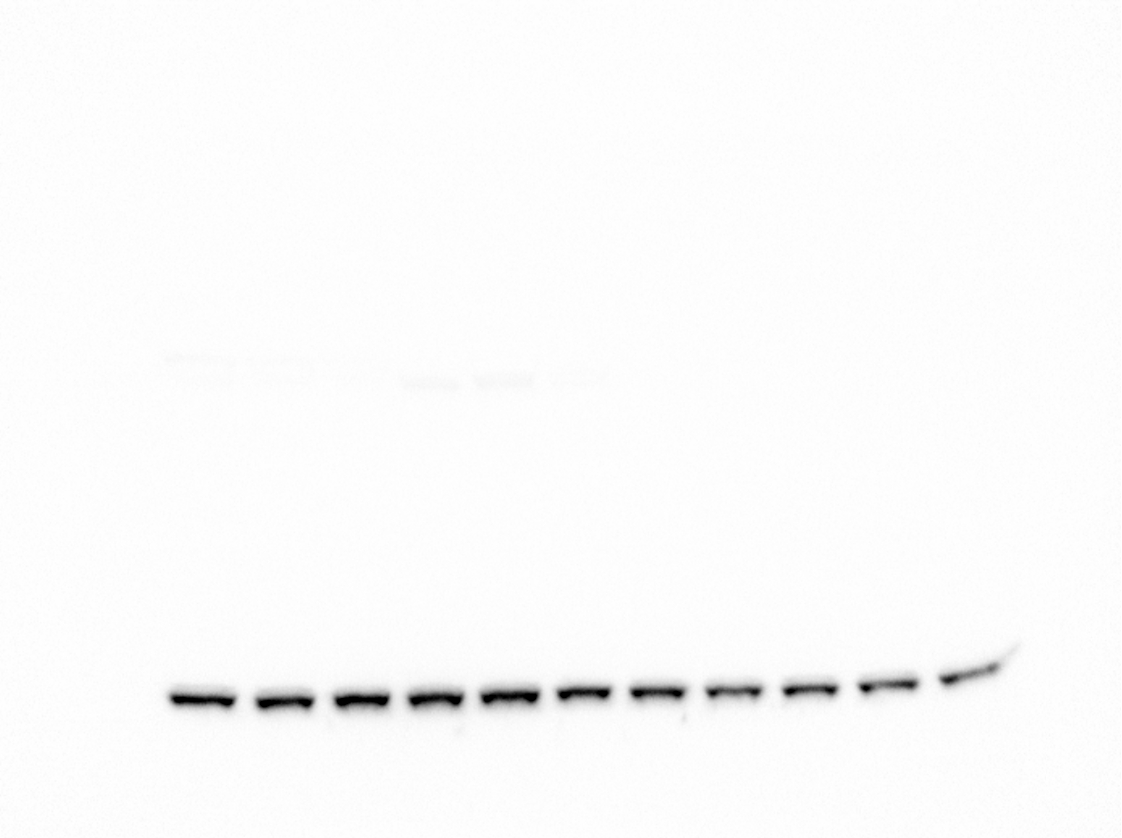

Supplement: Figure 5—figure supplement 3—source data 1. [file elife-106815-fig5-figsupp3-data1.zip › raw/Figure 5-figure supplement 3A_PSTAIRE.jpg]

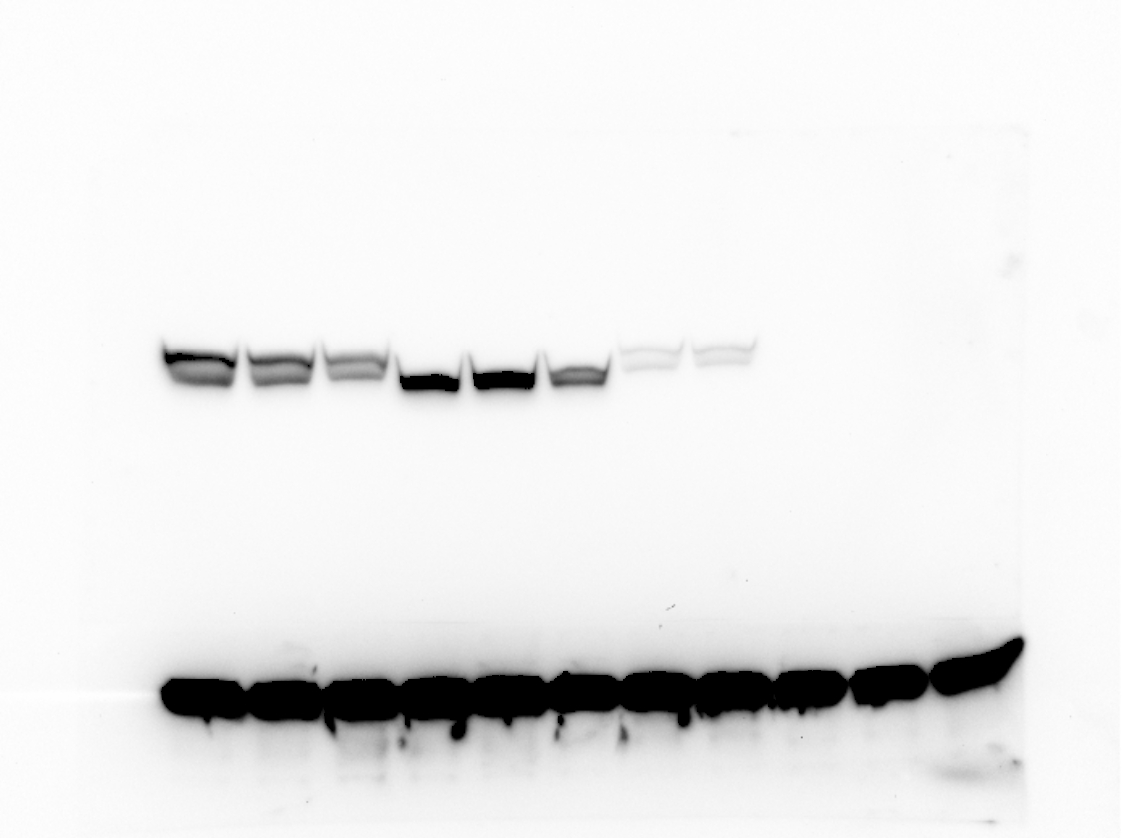

Supplement: Figure 5—figure supplement 3—source data 1. [file elife-106815-fig5-figsupp3-data1.zip › raw/Figure 5-figure supplement 3A_LT.jpg]

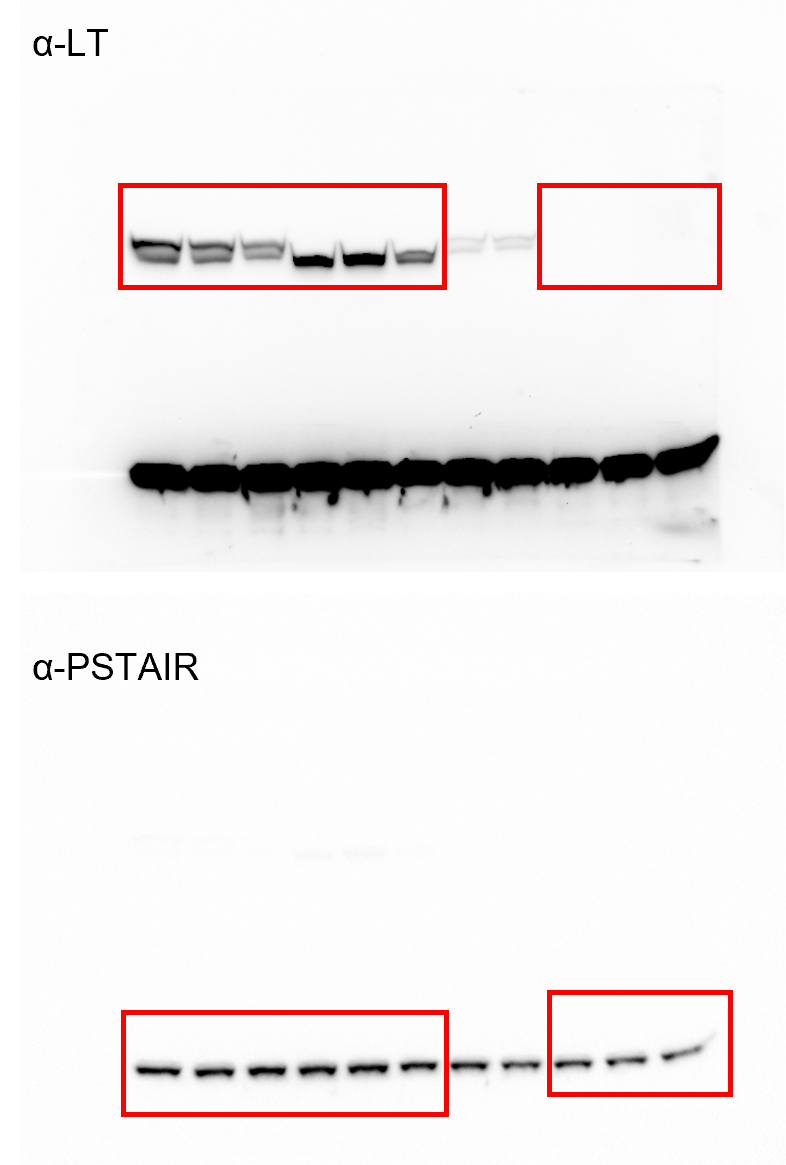

Supplement: Figure 5—figure supplement 3—source data 1. [file elife-106815-fig5-figsupp3-data1.zip › labelled/Figure 5-figure supplement 3A_labeled.tif]
